# Comparison of Serum Uric Acid and Serum Uric Acid‐to‐Creatinine Ratio as Indicators of Metabolic Syndrome: A Cross‐Sectional Study in China

**DOI:** 10.1002/edm2.70368

**Published:** 2026-11-06

**Authors:** Qiang Wang, Lanqiqi Wu, Ling Ma, Yin Cheng, Yining Liu, Jinyu Ma, Huitian Tong, Chao Shi

**Affiliations:** ^1^ People's Hospital of Ningxia Hui Autonomous Region Ningxia Medical University Yinchuan Ningxia Hui Autonomous Region China; ^2^ School of Public Health Ningxia Medical University Yinchuan Ningxia Hui Autonomous Region China; ^3^ Ningxia Clinical Research Institute People's Hospital of Ningxia Hui Autonomous Region Yinchuan Ningxia Hui Autonomous Region China

**Keywords:** adults, China, metabolic syndrome, serum uric acid, serum uric acid to creatine ratio

## Abstract

**Objective:**

This study aimed to systematically compare the roles of both serum uric acid (SUA) and Serum uric acid to creatine ratio (SUACr) as indicators of metabolic syndrome (MetS) in general populations.

**Methods:**

Information used here was gathered from a cross‐sectional investigation focusing on NHAR, about cardiovascular disease risk factors and prevalence. Dose–response relation between SUA, SUACr and MetS was evaluated using RCS model. Moreover, a logistic regression modelling was generated to evaluate the relation among SUA, SUACr and MetS within the total population.

**Results:**

A total of 10,217 subjects aged 18 and over were enrolled. Both SUA and SUACr were significantly associated with MetS and individual corresponding diseases in this population. Adjusted odds ratios indicated a progressive increase in the risk of MetS and individual corresponding diseases across quartiles of SUA and SUACr, all with the lowest quartile as reference for both genders (*p* for trend < 0.001). AUC of SUACr surpassed that for SUA in identifying MetS (0.61 vs. 0.59, *p* < 0.001). However, no statistical difference was observed between SUA and SUACr when stratified by gender. In men, the AUC of SUA and SUACr for MetS diagnosis were 0.61 and 0.62, respectively, while both SUA and SUACr had an AUC value of 0.62 in women.

**Conclusions:**

SUA and SUACr could serve as valuable predictors for early prevention, diagnosis and treatment of MetS in routine clinical practice. Upon gender stratification, there was no discernible statistical difference between SUA and SUACr in identifying MetS presence.

## Graphical abstract

Serum uric acid and its ratio to creatinine are both valuable predictors for the early prevention, diagnosis and treatment of metabolic syndrome in routine clinical practice.

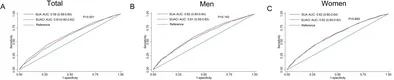

**Abbreviations**
DM: diabetes mellitus
FPG: fasting plasma glucose
HDL‐C: high‐density lipoprotein cholesterol
LDL‐C: low‐density lipoprotein cholesterol
MetS: Metabolic Syndrome
NHAR: Ningxia Hui Autonomous Region
SCr: serum creatinine
SUA: serum uric acid
SUACr: serum uric acid to creatinine ratio
TC: total cholesterol
TG: triglycerides

## 1 Introduction

Metabolic syndrome (MetS) encompasses lots of interconnected metabolic impairments, comprising obesity, central adiposity, mild dyslipidemia, hypertension and impaired glucose metabolism [1]. Recognized for its significant role in elevating the risk of diabetes, as well as cardiovascular mortality and morbidity [2]. MetS has seen a global increase in prevalence over recent decades [3]. Given the unclear etiological mechanisms underlying MetS, it is crucial to identify high‐risk populations and implement strategies to prevent the syndrome's progression and its associated negative health outcomes [4].

Uric acid, purine nucleotide metabolism end product, is primarily glomerulus‐filtered, then proximal tubule‐reabsorbed [5]. Link between MetS risk and serum uric acid (SUA) had been well established. Numerous researches indicated that hyperuricemia may forecast MetS onset [6, 7, 8]. Other research has found no significant correlation between SUA levels and MetS [9]. These conflicting conclusions might be due to the dependency of endogenous uric acid levels on the function of renal clearing [10]. As a result, serum uric acid‐to‐creatinine ratio (SUACr) served as a novel marker. This ratio is considered a better net uric acid production indicator, as it accounts for variations in renal function [11, 12].

However, a scarcity of studies that have simultaneously evaluated both SUA and SUACr as MetS indicators within the same population exists. Most prior research has focused on either SUA or SUACr independently and has predominantly involved subjects originating from healthcare centres or hospitals [13, 14, 15, 16]. These studies often involved individuals with existing comorbidities, such as diabetes, advanced age or an elevated risk of kidney and cardiovascular diseases. From a practical standpoint, there is a need to systematically compare the roles of both SUA and SUACr as indicators of MetS in general populations.

To fill the gaps, this study aims to explore the relation among SUA, SUACr and MetS in large sample research.

## 2 Materials and Methods

### 2.1 Study Population

Ningxia Cardiovascular Disorders Study (NCDS) performed a cross‐sectional investigation from January 2020 to December 2021 within the Ningxia Hui Autonomous Region (NHAR), China. The research aimed to explore the prevalence of cardiovascular diseases and their corresponding risk factors, including obesity, hypertension, diabetes, dyslipidemia, coronary heart disease and hyperuricemia [17]. This study utilized data from the aforementioned survey, which employed an extensive four‐stage stratified cluster sampling technique to obtain a representative general sample (aged 18 and above) in NHAR, China. Initially, nine counties within NHAR were selected in the first stage based on administrative and economic criteria (Figure S1). Second, two towns were randomly selected from each county using simple random sampling (SRS) from the local Center for Disease Control and Prevention. Subsequently, three villages or communities were randomly chosen from each selected town through SRS. Finally, as the fourth stage, samples were stratified according to gender distribution and age based on the 2010 China census data. The inclusion criteria were as follows: (1) age ≥ 18 years; (2) complete demographic information, physical examination and laboratory measurements; and (3) voluntary participation with written informed consent. The exclusion criteria comprised: (1) pregnant or lactating women; (2) missing data on key variables; and (3) presence of malignant tumours or end‐stage renal disease. A total of 10,217 subjects participated. After excluding 10 participants with incomplete data on serum uric acid, creatinine, or metabolic syndrome components, the final analytic sample comprised 10,207 individuals. Prior to the initiation of the survey, written informed consent was obtained from all participants, which included their consent to participate in the study and to allow the collected data to be used for future scientific research purposes.

### 2.2 Ethical Approval

Ethical approval was from the Institutional Review Board of the People's Hospital of Ningxia Hui Autonomous (2020‐YC‐002).

### 2.3 Data Collection and Anthropometry

All subjects used to visit the village health center or hospital in the morning period (with overnight fasting) for 8 h at least. Anthropometric measurements, comprising weight, waist circumference (WC) and height, were conducted following professional guidelines. Post resting for 5 min, sitting blood pressure was determined on the right arm utilizing a blood pressure electronic monitor (OMRON, HBP‐1120 U). Three readings were taken, and the mean value of the measuring results was documented to ensure accuracy. Additionally, well‐trained interviewers conducted computer‐assisted one‐on‐one private interviews to collect detailed information from the participants, including lifestyle factors, health status and socioeconomic status [17].

### 2.4 Blood Samples Collection and Biochemical Indicator Analyses

Each participant contributed around 5 mL blood sample after fasting, drawn into a tube containing heparin sodium as an anticoagulant. Tubes were subjected to centrifugation (1500 rpm, 10 min). After centrifugation, the resulting supernatants were carefully transferred into cryogenic vials then preserved at −80°C for further analysis. The supernatants were later sent to Beijing CIC Medical Laboratory for SUA, FPG, triglycerides, low‐density lipoprotein cholesterol (LDL‐C), serum creatinine (SCr), total cholesterol (TC) and high‐density lipoprotein cholesterol (HDL‐C) analyses. Quantification of LDL‐C, FPG, SUC, HDL‐C, TC, TG and SCr relied on AU 5800 device (Beckman Coulter Inc., California, USA) with reagents sourced from BiosinoBiotechnology and Science Inc., Beijing, China. Procedures were all conducted in strict compliance with pertinent protocols and regulatory standards.

Estimated glomerular filtration rate (eGFR) was computed by formula developed from the Chronic Kidney Disease Epidemiology Collaboration study:

$$\begin{array}{c} \text{eGFR}=141\times \min{\left(\mathrm{SCr}/k,1\right)}^{\alpha }\times \max{\left(\mathrm{SCr}/k,1\right)}^{-1.209}\\ \;\times 1.993^{\mathrm{age}}\times 1.018\;\left[\text{if female}\right] \end{array}$$

eGFR was computed using the following formula, with SCr represented by mg/dL, age by years. Here, “*k*” represents a coefficient specific to gender (0.7 for females, 0.9 for males), while “*α*” denotes constants (0.329 for females, 0.411 for males). The terms “max” and “min” denote SCr/k minimum or 1 and SCr/k maximum or 1, respectively.

### 2.5 Diagnostic Criteria

Referred from International Diabetes Federation (IDF), MetS is identified by central obesity (Chinese women: ≥ 80 cm, Chinese men: WC ≥ 90 cm), accompanied by abnormalities types counting ≥ 2: (1) Elevated TG > 1.7 mmol/L or receiving lipid impairment treating; (2) For women HDL‐C < 1.29 mmol/L, for men < 1.03 mmol/L, or receiving lipid impairment treating; (3) Diastolic blood pressure (DBP) ≥ 85 mmHg or SBP ≥ 130 mmHg, receiving treating for hypertension previously diagnosed; (4) FPG ≥ 5.6 mmol/L, or Type 2 diabetes previously diagnosed [18]. In all logistic regression models, each individual component (central obesity, hyperglycemia, elevated blood pressure, elevated triglycerides and reduced HDL‐C) as well as MetS (defined as central obesity combined with ≥ 2 of the above abnormalities) were treated as binary dependent variables (present vs. absent). The reference category for each binary outcome was defined as the absence of the condition.

### 2.6 Statistics

Descriptive statistics were computed for all metabolic and clinical variables. Categorical variables: percentages (%), continuous and normally distributed variables: Mean ± SD, skewed data: median (IQR). To compare baseline characteristics among different groups, categorical variables: *χ* ^2^ test; continuous variables: Kruskal–Wallis rank‐sum test/one‐way analysis of variance.

Multivariate and univariate logistic regressions were employed to compute OR for MetS referring to quartiles of SUA and SUACr. To examine dose–response relations between SUA/SUACr and MetS risk, restricted cubic splines (RCS) were employed with knots placed at the 10th, 50th and 90th percentiles. Reference: 10th percentiles (OR = 1).

The data were processed utilizing R software 3.2.2. Analyses utilized two‐sided tests, statistically significance: *p*‐values < 0.05.

Although our study employed a rigorous sampling design, methodological heterogeneity in epidemiological research—including differences in population characteristics, MetS definitions and laboratory protocols—may affect the comparability of results across studies. A harmonized methodological framework and standardized reporting are essential to ensure the reproducibility and generalizability of findings across diverse populations and settings [19].

## 3 Results

### 3.1 Characteristics of the Study Population

Table 1 displays laboratory outcomes and demographic features of subjects. Research comprised 10,207 subjects, including 4609 (45.11%) men and 5598 (54.89%) women, with median age as 44 years. MetS prevalence was higher among women (30.80%) compared to men (27.77%) (*p* < 0.001). MetS patients exhibited higher median age, average WC and body mass index (BMI) in comparison to those without MetS. MetS patients tended to have a higher percentage of people with a primary school education or less. They also exhibited higher blood pressure and elevated LDL‐C, TG, SUA and TC. Lower eGFR and HDL‐C were found within participants with MetS (*p* < 0.05).

**TABLE 1:** Selected characteristics and clinical characteristics in 10,207 individuals from Ningxia Hui Autonomous Region, China, 2020–2021.

Variables	Overall	No‐metabolic syndrome	Metabolic syndrome	*p*
	(*N* = 10,217)	(*N* = 7210)	(*N* = 3007)	
*Gender*				
Male	4609 (45.11)	3329 (46.17)	1280 (42.57)	< 0.05^a^
Female	5608 (54.89)	3881 (53.83)	1727 (57.43)	
*Age*				
Median (quartile)	44 (32–58)	41 (29–54)	53 (42–66)	
*Ethnic groups*, *n* (%)				
Han	7244 (70.90)	5106 (70.82)	2138 (71.10)	0.496^a^
Hui	2785 (27.26)	1964 (27.24)	821 (27.30)	
Others	188 (1.84)	140 (1.94)	48 (1.60)	
*Education*, *n* (%)				
Primary school or below	3478 (34.74)	2041 (28.31)	1437 (47.79)	< 0.001^a^
Middle school	2912 (28.50)	1993 (27.64)	919 (30.56)	
High school or above	3827 (37.46)	3176 (44.05)	651 (21.65)	
*Smoking*, *n* (%)				
No	7820 (76.54)	5511 (76.44)	2309 (76.79)	0.702
Yes	2397 (23.46)	1699 (23.56)	698 (23.21)	
*Drinking*, *n* (%)				
No	8040 (78.69)	5650 (78.36)	2390 (79.48)	0.209
Yes	2177 (21.31)	1560 (21.64)	617 (20.52)	
WC (cm), mean ± SD	83.06 ± 11.66	78.71 ± 9.93	93.51 ± 8.45	< 0.001^b^
BMI (kg/m^2^), mean ± SD	24.93 ± 3.91	23.67 ± 3.38	27.95 ± 3.44	< 0.001^b^
Hypertension, *n* (%)	3597 (35.21)	1661 (23.04)	1936 (64.38)	< 0.001^a^
Diabetes, *n* (%)	686 (6.71)	243 (3.37)	443 (14.73)	< 0.001^a^
SBP (mm Hg), mean ± SD	129.42 ± 19.73	124.40 ± 17.94	141.45 ± 18.58	< 0.001^b^
DBP (mm Hg), mean ± SD	80.74 ± 11.16	78.35 ± 10.41	86.47 ± 10.81	< 0.001^b^
HbA1c (%), mean ± SD	5.55 ± 0.60	5.45 ± 0.51	5.79 ± 0.73	< 0.001^b^
FPG (mmol/L), mean ± SD	5.56 ± 1.15	5.35 ± 0.90	6.06 ± 1.47	< 0.001^b^
TG (mmol/L), mean ± SD	1.47 ± 1.28	1.20 ± 0.96	2.12 ± 0.67	< 0.001^b^
TC (mmol/L), mean ± SD	4.26 ± 0.96	4.16 ± 0.91	4.50 ± 1.04	< 0.001^b^
HDL‐C (mmol/L), mean ± SD	1.26 ± 0.27	1.31 ± 0.28	1.15 ± 0.23	< 0.001^b^
LDL‐C (mmol/L), mean ± SD	2.58 ± 0.77	2.50 ± 0.74	2.77 ± 0.79	< 0.001^b^
SUA (mmol/L), mean ± SD	324.50 ± 90.46	315.57 ± 87.02	345.91 ± 94.86	< 0.001^b^
SCr (mmol/L), mean ± SD	65.64 ± 13.96	65.64 ± 13.96	65.34 ± 14.87	< 0.001^b^
SUACr, mean ± SD	5.03 ± 1.29	4.87 ± 1.21	5.40 ± 1.40	< 0.001^b^
eGFR (mL/min/1.73 m^2^), mean ± SD	107.60 ± 18.39	110.05 ± 18.00	101.71 ± 17.99	< 0.001^b^

Abbreviations: BMI, body mass index; DBP, diastolic blood pressure; eGFR, glomerular filtration rate; FPG, fasting plasma glucose; HbA1c, glycated haemoglobin; HDL‐C, high‐density lipoprotein cholesterol; LDL‐C, low‐density lipoprotein cholesterol; SBP, systolic blood pressure; SCr, serum creatinine; SUA, serum uric acid; SUACr, serum uric acid‐to‐creatinine ratio; TC, total cholesterol; TG, triglycerides; WC, waist circumference.

^a^ Indicates *p* values for categorical variables obtained using the chi‐squared (χ²) test, and superscript.

^b^ Indicates *p* values for continuous variables obtained using the Kruskal–Wallis rank‐sum test or one‐way analysis of variance.

### 3.2 Correlations Among SUA, SUACr With MetS and Individual Corresponding Diseases

Relations among SUA, SUACr and MetS with its constituting diseases are illustrated in Figure 1. In women, SUA was significantly correlated with MetS (*r* = 0.20, *p* < 0.001), WC (*r* = 0.17, *p* < 0.001), SBP (*r* = 0.10, *p* < 0.01), DBP (*r* = 0.09, *p* < 0.05), FPG (*r* = 0.11, *p* < 0.01), TG (*r* = 0.22, *p* < 0.001) and HDL‐C (*r* = −0.08, *p* < 0.05). In men, SUA showed significant correlations with MetS (*r* = 0.19, *p* < 0.001), WC (*r* = 0.14, *p* < 0.001), TG (*r* = 0.20, *p* < 0.001) and HDL‐C (*r* = −0.10, *p* < 0.01), but not with FPG (*r* = 0.00, *p* = 0.97) or SBP/DBP (*p* > 0.05). Similar correlation patterns were observed between SUACr and MetS as well as its components. Specifically, in women, SUACr was significantly correlated with MetS (*r* = 0.21, *p* < 0.001), WC (*r* = 0.18, *p* < 0.001), SBP (*r* = 0.09, *p* < 0.01), DBP (*r* = 0.08, *p* < 0.05), FPG (*r* = 0.12, *p* < 0.01), TG (*r* = 0.23, *p* < 0.001) and HDL‐C (*r* = −0.09, *p* < 0.01); in men, SUACr was significantly correlated with MetS (*r* = 0.19, *p* < 0.001), WC (*r* = 0.15, *p* < 0.001), TG (*r* = 0.21, *p* < 0.001) and+ HDL‐C (*r* = −0.11, *p* < 0.01), but not with FPG (*r* = 0.01, *p* = 0.85) or SBP/DBP (*p* > 0.05).

**FIGURE 1:**
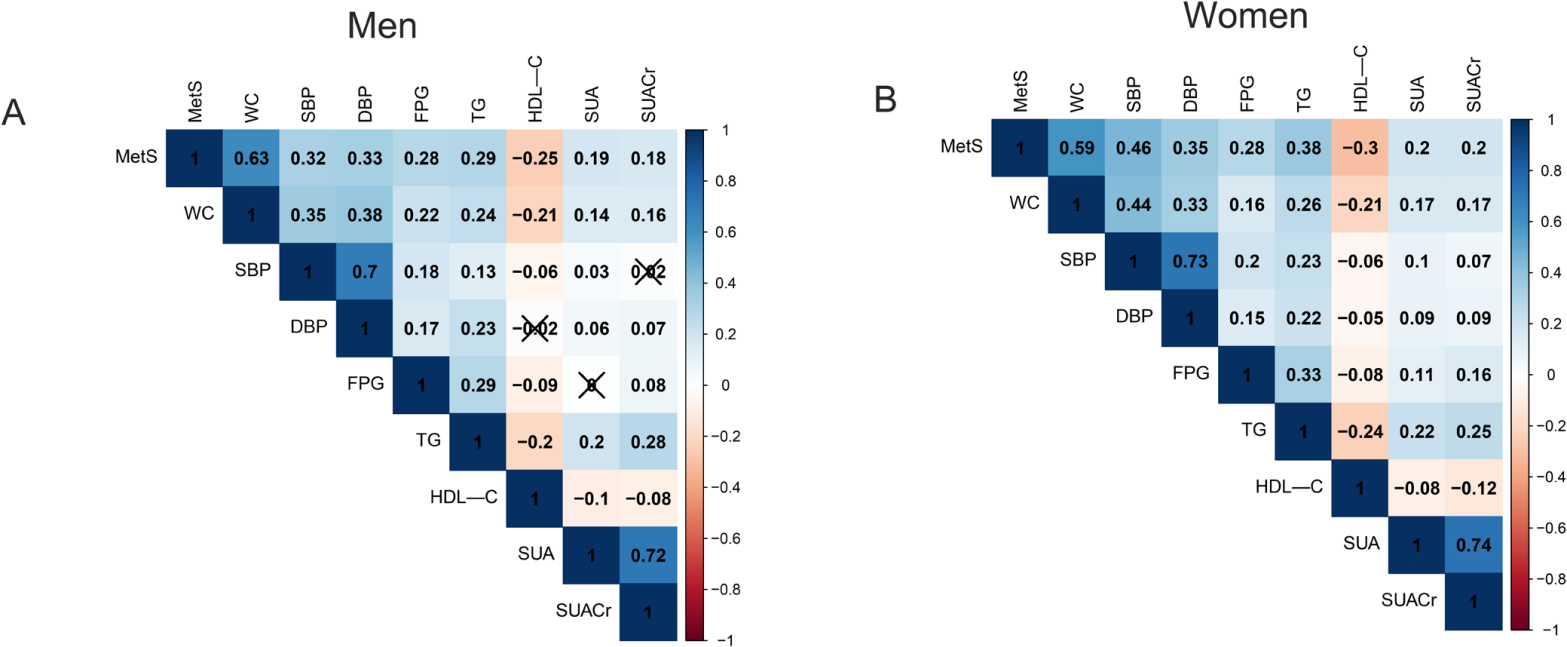
Relations among serum uric acid, serum uric acid‐to‐creatinine ratio and metabolic syndrome with its constituting diseases. **×:** No statistical difference. DBP, diastolic blood pressure; FPG, fasting plasma glucose; HDL‐C, high‐density lipoprotein cholesterol; MetS, metabolic syndrome; SBP, systolic blood pressure; SUA, serum uric acid; SUACr, serum uric acid‐to‐creatinine ratio; TG, triglycerides; WC, waist circumference.

Moreover, both SUA and SUACr levels exhibited an increase corresponding to the count of MetS risk factors within both genders (Figure 2).

**FIGURE 2:**
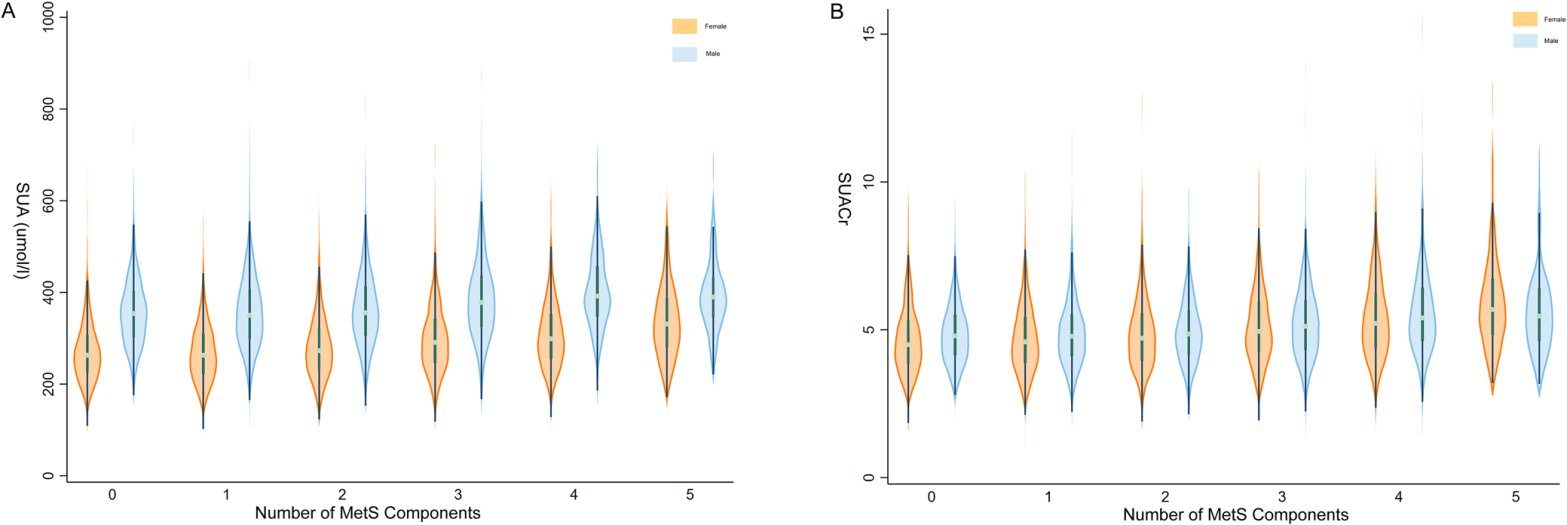
Associations between serum uric acid, serum uric acid‐to‐creatinine levels and the number of metabolic syndrome components. MetS, metabolic syndrome; SUA, serum uric acid; SUACr, serum uric acid‐to‐creatinine ratio.

### 3.3 Dose–Response Relation Between SUA, SUACr and MetS

Figure 3 illustrates the dose–response relationships assessed using the RCS model. Among men, both SUA (*p*‐overall < 0.001 and *p*‐non‐linear < 0.05) and SUACr (*p*‐overall < 0.001 and *p*‐non‐linear < 0.05) exhibited a non‐linear relationship and an elevating trend with MetS prevalence. Conversely, among women, SUA (*p*‐overall < 0.001 and *p*‐non‐linear = 0.585) and SUACr (*p*‐overall < 0.001 and *p*‐non‐linear = 0.248) exhibited a non‐linear relation with MetS prevalence.

**FIGURE 3:**
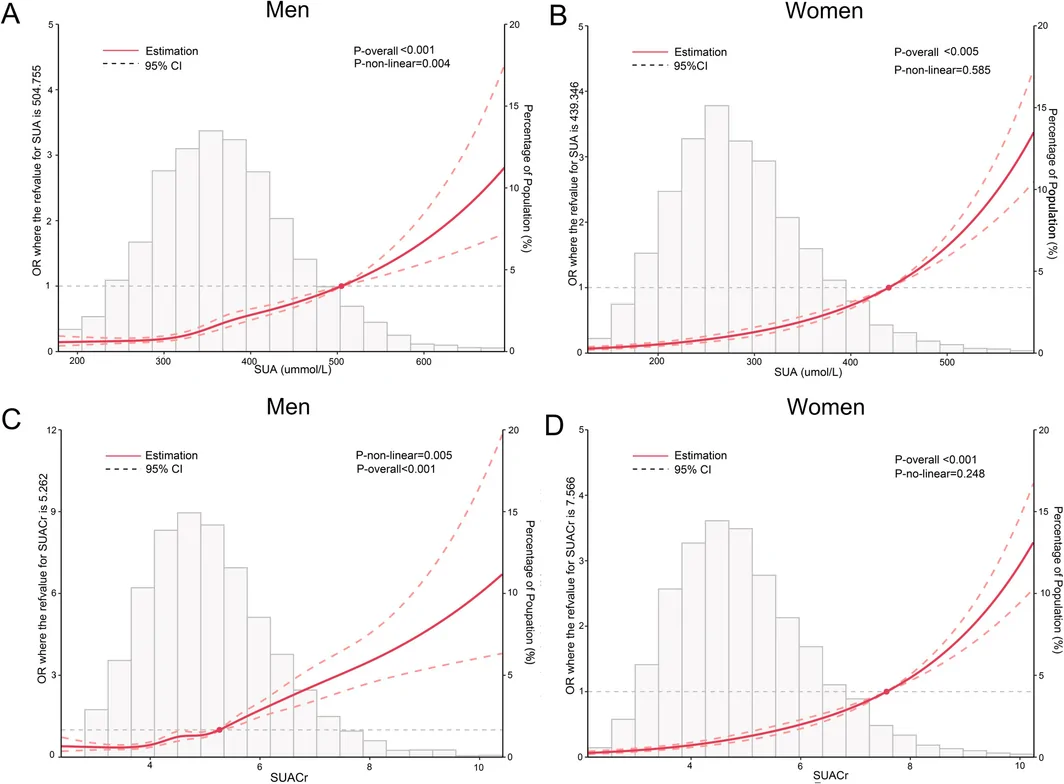
The restricted cubic spline for the relationships between levels of serum uric acid, serum uric acid‐to‐creatinine ratio and the risk of metabolic syndrome. (A, B) The restricted cubic spline for the relationships between levels of SUA and the risk of MetS. (C, D) The restricted cubic spline for the relationships between levels of SUACr and the risk of MetS. Adjusted confounders were for age, ethnic groups, education, smoking, drinking and eGFR. MetS, metabolic syndrome; SUA, serum uric acid; SUACr, serum uric acid‐to‐creatinine ratio.

### 3.4 Logistic Regression Analyses on SUA, SUACr With MetS and Corresponding Constituting Diseases

Significant interactions were observed between SUA and gender (*p*‐interaction < 0.001) as well as between SUACr and gender (*p*‐interaction < 0.001). Accordingly, all subsequent logistic regression analyses were performed separately for men and women (refer to Tables 2 and 3). After adjusting for smoking, age, drinking, education and eGFR, both SUA and SUACr were observed to positively associate with MetS and its constituting diseases risk within both genders. Adjusted ORs for the risk increased with quartiles of SUA and SUACr in comparison to the lowest quartile in both genders (*p* for trend < 0.001).

**TABLE 2:** Logistic regression analysis of MetS and its components by SUACr quartile in 10,207 individuals from Ningxia Hui Autonomous Region, China, 2020–2021.

Variable	Men					Women				
	Q1 (1.58–4.18)	Q2 (4.18–4.96)	Q3 (4.97–5.90)	Q4 (5.91–15.29)	*p* for trend^a^	Q1 (1.32–3.96)	Q2 (3.97–4.84)	Q3 (4.85–5.90)	Q4 (5.91–12.94)	*p* for trend^a^
*MetS*										
Crude OR (95% CI)	1.00	1.35 (1.10–1.65)	1.76 (1.45–2.15)	2.83 (2.32–3.44)	< 0.001	1.00	1.58 (1.33–1.89)	1.97 (1.65–2.34)	3.17 (2.66–3.79)	< 0.001
Adjusted OR (95% CI)^b^	1.00	1.72 (1.40–2.12)	2.72 (2.20–3.37)	5.81 (4.61–7.32)	< 0.001	1.00	1.74 (1.44–2.11)	2.60 (2.14–3.17)	5.68 (4.59–7.03)	< 0.001
*Central obesity*										
Crude OR (95% CI)	1.00	1.32 (1.11–1.57)	1.62 (1.36–1.92)	2.33 (1.95–2.78)	< 0.001	1.00	1.39 (1.20–1.61)	1.54 (1.33–1.79)	2.15 (1.83–2.53)	< 0.001
Adjusted OR (95% CI)^b^	1.00	1.64 (1.37–1.97)	2.41 (2.00–2.91)	4.49 (3.64–5.54)	< 0.001	1.00	1.60 (1.35–1.88)	2.14 (1.80–2.54)	3.97 (3.26–4.83)	< 0.001
*Hyperglycemia*										
Crude OR (95% CI)	1.00	1.12 (0.95–1.32)	1.25 (1.05–1.48)	1.55 (1.30–1.84)	< 0.001	1.00	1.24 (1.06–1.45)	1.49 (1.27–1.75)	1.94 (1.65–2.30)	< 0.001
Adjusted OR (95% CI)^b^	1.00	1.29 (1.08–1.53)	1.59 (1.33–1.90)	2.25 (1.84–2.74)	< 0.001	1.00	1.25 (1.06–1.47)	1.56 (1.32–1.85)	2.20 (1.83–2.64)	< 0.001
*Elevated BP*										
Crude OR (95% CI)	1.00	1.04 (0.88–1.22)	1.00 (0.85–1.19)	1.20 (1.01–1.43)	0.049	1.00	1.21 (1.05–1.41)	1.34 (1.15–1.56)	1.52 (1.29–1.78)	< 0.001
Adjusted OR (95% CI)^b^	1.00	1.52 (1.26–1.82)	1.85 (1.53–2.24)	3.00 (2.42–3.72)	< 0.001	1.00	1.28 (1.07–1.53)	1.68 (1.39–2.03)	2.47 (2.00–3.04)	< 0.001
*Elevated TG*										
Crude OR (95% CI)	1.00	1.50 (1.24–1.82)	1.91 (1.58–2.31)	3.39 (2.80–4.10)	< 0.001	1.00	1.45 (1.18–1.79)	2.33 (1.91–2.86)	4.18 (3.41–5.12)	< 0.001
Adjusted OR (95% CI)^b^	1.00	1.67 (1.37–2.04)	2.41 (1.97–2.96)	5.38 (4.31–6.32)	< 0.001	1.00	1.59 (1.28–1.97)	2.93 (2.37–3.63)	6.30 (5.04–7.89)	< 0.001
*Low HDL‐C*										
Crude OR (95% CI)	1.00	1.12 (0.93–1.35)	1.10 (0.91–1.33)	1.49 (1.23–1.80)	< 0.001	1.00	1.19 (1.03–1.38)	1.40 (1.21–1.63)	1.77 (1.51–2.08)	< 0.001
Adjusted OR (95% CI)^b^	1.00	1.21 (1.00–1.47)	1.24 (1.02–1.52)	1.80 (1.45–2.22)	< 0.001	1.00	1.25 (1.08–1.46)	1.55 (1.33–1.82)	2.05 (1.72–2.43)	< 0.001

*Note:* The reference category for all odds ratios (ORs) was the lowest quartile of SUA/SUACr, and the reference category for the outcome variables was the absence of the corresponding disease.

Abbreviations: MetS, metabolic syndrome; SUA/Cr, serum uric acid to creatinine ratio.

^a^ Tests for linear trend were conducted by using the median of each quartile category of SUACr as a continuous variable, adjusting forage, ethnic groups, education, smoking, drinking and eGFR.

^b^ Model was adjusted for age, ethnic groups, education, smoking, drinking and eGFR.

**TABLE 3:** Logistic regression analysis of MetS and its components by SUA quartile in 10,207 individuals from Ningxia Hui Autonomous Region, China, 2020–2021.

Variable	Men					Women				
	Q1 (124–312)	Q2 (313–365)	Q3 (366–421)	Q4 (422–889)	*p* for trend^a^	Q1 (71–234)	Q2 (235–277)	Q3 (278–327)	Q4 (328–705)	*p* for trend^a^
*MetS*										
Crude OR (95% CI)	1.00	1.50 (1.22–1.84)	2.21 (1.81–2.69)	3.03 (2.50–3.67)	< 0.001	1.00	1.41 (1.18–1.68)	1.90 (1.60–2.25)	2.95 (2.50–3.49)	< 0.001
Adjusted OR (95% CI)^b^	1.00	1.76 (1.43–2.17)	3.12 (2.53–3.84)	5.68 (4.57–7.07)	< 0.001	1.00	1.46 (1.21–1.76)	2.21 (1.83–2.67)	4.53 (3.72–5.53)	< 0.001
*Central obesity*										
Crude OR (95% CI)	1.00	1.61 (0.98–1.39)	1.62 (1.37–1.93)	2.27 (1.91–2.69)	< 0.001	1.00	1.39 (1.20–1.61)	1.69 (1.45–4.96)	2.20 (1.89–2.56)	< 0.001
Adjusted OR (95% CI)^b^	1.00	1.35 (1.13–1.61)	2.23 (1.86–2.68)	4.12 (3.39–5.02)	< 0.001	1.00	1.51 (1.28–1.78)	2.08 (1.76–2.47)	3.55 (2.95–4.27)	< 0.001
*Hyperglycemia*										
Crude OR (95% CI)	1.00	1.13 (0.96–1.34)	1.31 (1.11–1.55)	1.40 (1.18–1.65)	< 0.001	1.00	1.09 (0.92–1.27)	1.30 (1.11–1.52)	1.82 (1.56–2.13)	< 0.001
Adjusted OR (95% CI)^b^	1.00	1.27 (1.06–1.50)	1.65 (1.38–1.97)	2.05 (1.71–2.47)	< 0.001	1.00	1.06 (0.90–1.25)	1.32 (1.12–1.55)	1.95 (1.65–2.31)	< 0.001
*Elevated BP*										
Crude OR (95% CI)	1.00	1.12 (0.95–1.31)	1.22 (1.04–1.44)	1.14 (0.96–1.34)	0.094	1.00	1.13 (0.97–1.32)	1.41 (1.21–1.63)	1.71 (1.47–1.98)	< 0.001
Adjusted OR (95% CI)^b^	1.00	1.47 (1.23–1.76)	2.04 (1.69–2.46)	2.49 (2.04–3.03)	< 0.001	1.00	1.05 (0.87–1.26)	1.43 (1.19–1.72)	2.10 (1.72–2.55)	< 0.001
*Elevated TG*										
Crude OR (95% CI)	1.00	1.62 (1.33–1.96)	2.19 (1.82–2.65)	3.61 (3.00–4.35)	< 0.001	1.00	1.84 (1.49–2.27)	2.37 (1.93–2.92)	4.61 (3.79–5.62)	< 0.001
Adjusted OR (95% CI)^b^	1.00	1.71 (1.40–2.09)	2.64 (2.16–3.22)	5.33 (4.33–6.58)	< 0.001	1.00	1.92 (1.54–2.38)	2.59 (2.09–3.21)	5.98 (4.83–7.40)	< 0.001
*Low HDL‐C*										
Crude OR (95% CI)	1.00	1.27 (1.05–1.53)	1.38 (1.15–1.66)	1.22 (1.01–1.47)	0.038	1.00	1.03 (0.89–1.19)	1.17 (1.01–1.36)	1.50 (1.30–1.74)	< 0.001
Adjusted OR (95% CI)^b^	1.00	1.40 (1.16–1.70)	1.65 (1.36–2.01)	1.67 (1.36–2.04)	< 0.001	1.00	1.11 (0.95–1.29)	1.38 (1.19–1.61)	1.97 (1.68–2.32)	

*Note:* The reference category for all odds ratios (ORs) was the lowest quartile of SUA/SUACr, and the reference category for the outcome variables was the absence of the corresponding disease.

Abbreviations: MetS, metabolic syndrome; SUA, serum uric acid.

^a^ Tests for linear trend were conducted by using the median of each quartile category of SUA/Cr as a continuous variable, adjusting for age, ethnic groups, education, smoking, drinking and eGFR.

^b^ Model was adjusted for age, ethnic groups, education, smoking, drinking and eGFR.

### 3.5 Distinguishing Between SUA and SUACr for the Identification of MetS

Figure 4 displays AUC values for SUA and SUACr in identifying the presence of MetS. As shown in Figure 4, in terms of identifying metabolic syndrome (MetS), the area under the curve (AUC) of SUACr was higher than that of SUA (0.61 vs. 0.59, *p* < 0.001). However, no statistical difference was observed between SUA and SUACr after stratifying by gender. In men, the AUC values for SUA and SUACr in MetS diagnosis were 0.61 and 0.62, respectively, while both SUA and SUACr had an AUC of 0.62 in women.

**FIGURE 4:**
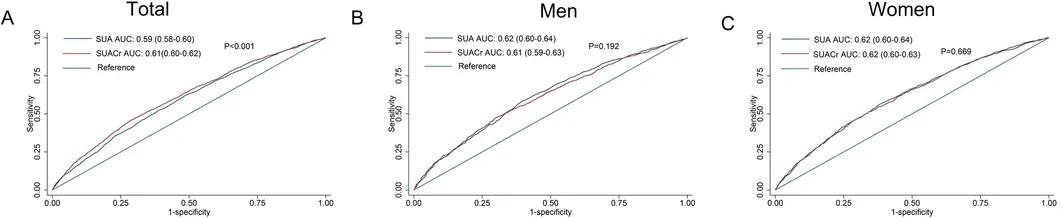
Receiver operating characteristic curve for serum uric acid and serum uric acid‐to‐creatinine ratio in identifying the presence of metabolic syndrome. AUC, area under curve; MetS, metabolic syndrome; SUA, serum uric acid; SUACr, serum uric acid‐to‐creatinine ratio.

## 4 Discussion

This study systematically investigated the role of SUA and SUACr as MetS indicators in a general population aged 18 years and above. Our findings demonstrated significant relations of both SUA and SUACr with MetS and its constituting diseases, indicating their potential as predictive biomarkers for assessing MetS risk. However, there was no statistical difference between SUA and SUACr in identifying the presence of MetS after stratifying by gender. To our knowledge, it's the first study that simultaneously investigate SUAand SUACr as MetS indicators within the same general population in China. The findings highlight the potential for these biomarkers to aid in the early identification of individuals at high MetS risk.

Link between MetS risk and SUA has long been established. In follow‐up research continuing 2.6 years involving 1590 adults without MetS (929 women and 661 men) aged 40–70 years, SUA baseline levels were notably related to an increased risk of developing high WC, BP, TG, as well as developing MetS [8]. However, other retrospective researches have not stably shown a strong relation from SUA to MetS, with some failing to find significant relations with MetS components as well [9]. These discrepancies might be attributed to renal clearance impairment, where subjects with lower eGFRs tend to have greater concentrations of uric acid. Thus, SUACr, as a measure normalized for renal function, may provide a more accurate reflection of net uric acid production [20].

Numerous studies explored link from SUACr to MetS. For example, within prospective cohort involving 1072 community residents, Japanese researchers found that SUACr baseline exhibited independent and significant relation with MetS after follow‐up period lasting 3 years [21]. Al‐Daghri et al. [22] also documented that subjects in SUACr's highest quartile showed 1.80 times higher MetS risk in comparison to those in the lowest quartile. Similarly, in line with previous findings, our study showed adjusted ORs and 95% CIs of 2.67 (1.50–4.74) for SUACr's highest quartile in comparison to the lowest quartile, highlighting significant associations with MetS and constituting diseases.

Following adjustment for smoking, drinking, education, age and eGFR, we observed that both SUA and SUACr were positively related to hyperglycemia, elevated blood pressure, elevated TC and central obesity in both genders. However, findings from Tengrui Cao et al. [13] indicated a significant positive correlation between SUACr and the aforementioned metabolic abnormalities, with no significant association observed with low HDL‐C. The inconsistent results between the two studies may be attributed to differences in sampling methods: the previous study focused on individuals aged 45 years and above, whereas this study included all age groups from 18 years onward.

The exact mechanisms linking SUA and MetS are not yet fully understood. Possible pathways through which SUA may be associated with the development of MetS include its potential to suppress lipoprotein enzymes involved in lipid metabolism, thereby influencing adipokines that regulate fat synthesis [23]. Moreover, SUA has been implicated in the activation of the renin‐angiotensin system, which may reduce renal blood flow, induce endothelial impairment and promote vascular smooth muscle cell proliferation throughout the systemic circulation [24]. These effects might play a role in the progression of hypertension. Additionally, SUA is thought to be related to oxidative stress within adipocytes through elevated levels of monocyte chemoattractant protein‐1 and reduced adiponectin, which may be involved in adipogenesis and insulin resistance [25].

Emerging evidence suggests that the interplay between chronic inflammation and metabolic dysregulation in MetS is partially mediated by immunometabolic reprogramming. Alterations in mitochondrial metabolism and autophagic flux in tissue‐resident macrophages can shift the balance between pro‐inflammatory and reparative phenotypes, thereby participating in the development of insulin resistance, adipose tissue dysfunction, and the progression of metabolic abnormalities. This immunometabolic framework may help to explain the observed associations between uric acid and MetS components [26].

Previous researches have indicated role of sex in relation between SUA and MetS [15, 27], confirmed in our study. Our results indicated significant interactions between SUA/SUACr, and gender. No statistical difference was observed between SUA and SUACr after stratifying by gender. The mechanisms underlying these gender differences are likely related to the protective effects of oestrogen on uric acid metabolism and insulin sensitivity. Oestrogen has been demonstrated to enhance renal uric acid excretion and suppress xanthine oxidase activity, which may explain the stronger association between SUA and MetS observed in postmenopausal women [28, 29]. In addition, oestrogen regulates adipose tissue distribution and inflammatory responses, potentially mitigating the adverse metabolic effects of uric acid in premenopausal women [30]. Our findings are consistent with previous studies reporting sex‐specific associations [15, 27]. Nevertheless, study findings have potential implications for evaluating MetS risk in clinical practice. Considering the application of SUA and SUACr as markers in MetS screening, SUA is recommended due to its simplicity and lower cost compared to SUACr. Given the modest discriminatory capacity of any single biomarker, future strategies for risk stratification in metabolic syndrome may be enhanced by adopting a multidimensional assessment framework—one that incorporates molecular biomarkers, anthropometric parameters and functional measures into a coherent model. In particular, combining SUA or SUACr with readily available clinical indices, such as waist circumference and blood pressure, may offer a more comprehensive and practically applicable diagnostic approach than reliance on any individual marker alone [31]. Primary advantages in the research comprised design relying on population employing broad age distribution, large sample size, and random sampling, which ensured the generalizability. Additionally, comprehensive data on potential confounders enabled multivariable adjustment, providing a detailed examination of relations between MetS and SUA/SUACr. Nevertheless, limitations exist. First, the cross‐sectional design precludes any causal inference regarding the associations between SUA/SUACr and MetS. Secondly, serum uric acid and creatinine were measured only once, which may not adequately reflect long‐term exposure levels and is subject to intra‐individual variability. Third, the use of IDF criteria alone to define MetS may limit comparability with studies adopting alternative definitions, such as ATP III or harmonized criteria. Fourth, although we adjusted for multiple confounders, residual confounding from unmeasured variables—including dietary intake, physical activity, medication use (e.g., diuretics, allopurinol) and genetic factors—cannot be entirely excluded. Fifth, the sample was drawn from a single autonomous region in China, which may restrict the generalizability of our findings to other populations.

## 5 Conclusions

SUA and SUACr serve as valuable predictors for early prevention, diagnosis and MetS treatment in routine clinical practice. Upon stratification by gender, no statistically discernible distinction exists between SUA and SUACr in identifying the presence of MetS. Given the challenges associated with detection, relying on SUA alone as a diagnostic indicator may be more practical; however, depending solely on a single marker has limited efficacy. It is essential to integrate other indicators to construct a comprehensive diagnostic model.

## Author Contributions

**Qiang Wang:** writing – original draft, formal analysis. **Lanqiqi Wu:** writing – original draft, formal analysis. **Ling Ma:** investigation, formal analysis. **Yin Cheng:** investigation, formal analysis. **Yining Liu:** investigation. **Jinyu Ma:** investigation. **Huitian Tong:** investigation. **Chao Shi:** conceptualization, methodology, data curation, writing – original draft, formal analysis, supervision.

## Funding

This work was supported by the Open Research Fund of Ningxia Clinical Research Institute, People's Hospital of Ningxia Hui Autonomous Region, the Natural Science Foundation of Ningxia (No. 2023AAC05055).

## Ethics Statement

Research protocols of this study were approved by the Institutional Review Board of the People's Hospital of Ningxia Hui Autonomous, where the work was undertaken and that it conforms to the provisions of the Declaration of Helsinki (as revised in Forta‐leza, Brazil, October 2013). Registry and the registration no. of the study/trial: March 18, 2020 (No. 2020‐YC‐002).

## Consent

All participants provided written informed consent.

## Conflicts of Interest

The authors declare no conflicts of interest.

## Supporting information


**Figure S1:** Distribution of study area for Ningxia Cardiovascular Disorders Study (NCDS), 2020–2021.

## Data Availability

The datasets analysed during this study are not publicly available but are available from the corresponding author on reasonable request.
