# Supplementary material for: Comparison of Serum Uric Acid and Serum Uric Acid‐to‐Creatinine Ratio as Indicators of Metabolic Syndrome: A Cross‐Sectional Study in China
Source: Endocrinol Diabetes Metab. 2026 Nov 6;9(6):e70368. doi: 10.1002/edm2.70368 (PMC13743622; doi:10.1002/edm2.70368)
Supplement: Supplementary file 1 [file EDM2-9-e70368-s001.docx]

Supplementary Material

# Supplementary Figures

**Supplementary Figure 1** Distribution of study area for Ningxia Cardiovascular Disorders Study (NCDS), 2020-2021.
